# Network pharmacological identification of active compounds and potential actions of *Erxian decoction* in alleviating menopause-related symptoms

**DOI:** 10.1186/s13020-015-0051-z

**Published:** 2015-07-19

**Authors:** Shiwei Wang, Yao Tong, Tzi-Bun Ng, Lixing Lao, Jenny Ka Wing Lam, Kalin Yanbo Zhang, Zhang-Jin Zhang, Stephen Cho Wing Sze

**Affiliations:** School of Chinese Medicine, Li Ka Shing Faculty of Medicine, The University of Hong Kong, Hong Kong SAR, China; School of Biomedical Sciences, Faculty of Medicine, The Chinese University of Hong Kong, Hong Kong SAR, China; Department of Pharmacology and Pharmacy, Li Ka Shing Faculty of Medicine, The University of Hong Kong, Hong Kong SAR, China

## Abstract

**Background:**

*Erxian decoction* (EXD) is used to treat menopause-related symptoms in Chinese medicine. This study aims to identify the bioactive compounds and potential actions of EXD by network pharmacological analysis.

**Methods:**

Two databases, the Traditional Chinese Medicine Systems Pharmacology database and TCM Database@Taiwan, were used to retrieve literature of phytochemicals of EXD. STITCH 4.0 and the Comparative Toxicogenomics Database were used to search for compound–protein and compound–gene interactions, respectively. DAVID Bioinformatics Resources 6.7 and Cytoscape 3.01 with Jepetto plugin software were used to perform a network pharmacological analysis of EXD.

**Results:**

A total of 721 compounds were identified in EXD, of which 155 exhibited 2,656 compound–protein interactions with 1,963 associated proteins determined by STITCH4.0 database, and of which 210 had 14,893 compound–gene interactions with 8,536 associated genes determined by Comparative Toxicogenomics Database. Sixty three compounds of EXD followed the Lipinski’s Rule with OB ≥30% and DL index ≥0.18, of which 20 related to 34 significant pathway- or 12 gene- associated with menopause.

**Conclusions:**

Twenty compounds were identified by network pharmacology as potential effective ingredients of EXD for relieving menopause with acceptable oral bioavailability and druggability.

**Electronic supplementary material:**

The online version of this article (doi:10.1186/s13020-015-0051-z) contains supplementary material, which is available to authorized users.

## Background

By the age of 35 years, the quality and quantity of ovarian follicles would decline [1], and consequential hormonal and symptomatic changes would lead to cessation of menses [2]. During menopause, the fluctuating levels of sex hormones, including luteinizing hormone, follicle-stimulating hormone, estrogen, and progesterone [3], can cause osteoporosis and menopausal symptoms, such as hot flushes, depression, nocturnal sweating, uterine bleeding, vaginal dryness, insomnia, and loss of sexual function [4–6]. It is estimated that there will be about 1.2 billion menopausal women worldwide by 2030 [7]. Menopause occurs between 44.6 and 52 years of age, varying among different races and countries [8]. In the United States, about 6,000 women reach menopause every day, which is more than 2 million per year [7]. The average age of menopause in the United Kingdom and United States is 52 and 51 years, respectively [9, 10]. In China, women around 50 years of age would experience natural menopause and in the southeast of China reach menopause at an average age of 48.9 years [11, 12]; thus, 0.28 billion women will be over the age of 50 years by 2030 would have menopause [13].

Hormone replacement therapy (HRT) has been used for more than 60 years to relieve menopausal symptoms. However, there are many adverse effects associated with HRT [14], e.g., increasing the risks of breast cancer, coronary artery disease, endometrial cancer, venous thromboembolism and stroke [15].

Chinese medicines (CM) are also used in treating menopausal symptoms [16–21]. Some Chinese herbal formulas (CHFs) are indicative for treating gynecological disorders including menopausal symptoms [16, 17]. However, few studies on the biological actions of the CHFs have been conducted [24–26]. As a typical example for CHFs, *Erxian decoction* (EXD) is commonly used to treat menopause related symptoms [17, 22–34], consisting of six herbs, Herba *Epimedium Brevicornum* (HE; *Xian*-*ling*-*pi*), Rhizoma *CurculiginisOrchioides* (RC; *Xian*-*mao*), Radix *Morindae Officinalis* (RMO; *Ba*-*ji*-*tian*), Radix *Angelicae Sinensis* (RAS; *Dang*-*gui*), Cortex *Phellodendri Chinensis* (CPC; *Huang*-*bo*), and Rhizoma *AnemarrhenaeAsphodeloides* (RA; *Zhi*-*mu*) [35].

During the past two decades, drug discovery has pursued a dominant target, “one drug, one disease” paradigm. However, many drugs exert therapeutic effects via restoration of multiple disease-related targets rather than a single one [36, 37]. Network pharmacology, which is based on systems biology, polypharmacology and molecular network analysis, provides a possible strategy to elucidate the action mechanism of multi-ingredient medicine in a holistic view [38–40]. Molecular networks are constructed by interactions of target-based proteins and genes for predicting their function and facilitating drug discovery, which provides pharmacological information in a holistic manner [40, 41]. Enrichment analysis is an analytical method to assess functional associations between sets of genes or proteins of interest to us and a database of known gene or protein sets [42, 43]. It can identify the significant pathways and their enriched gene/protein sets, and elucidate significant multiple pharmacological mechanisms [42, 44].

The complexity of numerous chemical constituents and biological actions has not been fully identified in EXD. This study aims to identify the bioactive compounds and actions of EXD by a network pharmacological analysis.

## Methods

**Figure 1:**
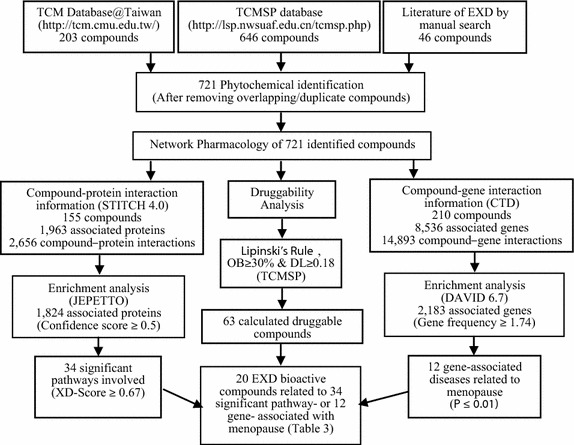
The workflow of the network pharmacological study of EXD.

The constituent compounds of EXD were identified by two phytochemical databases, the Traditional Chinese Medicine Systems Pharmacology (TCMSP) database and TCM Database@Taiwan., as well as published EXD literatures [26–30, 35, 45, 46]. The druggability analysis of the identified compounds in EXD were performed and provided by Lipinski’s rule (LR) and TCMSP database in term of oral bioavailability (OB) and drug-likeness (DL) indices, respectively. OB is the degree to which a drug or other substance becomes available to the target tissue after oral administration. DL is to evaluate their potentials to be bioactive compounds compare with the well-developed drug. The significant pathways and gene-associated diseases for the identified compounds were determined by enrichment analysis (JEPETTO (US): http://apps.cytoscape.org/apps/jepetto) [43] of the compound-protein interaction and enrichment analysis (DAVID 6.7 (US): http://david.abcc.ncifcrf.gov/home.jsp) [47] of the compound–gene interactions, respectively. The workflow of the network pharmacology study of EXD was summarized in Figure 1.

### Identification of potential bioactive constituents in EXD

All phytochemicals from the six constituent herbs of EXD were identified by the TCM Database@Taiwan (http://tcm.cmu.edu.tw/), TCMSP database (http://sm.nwsuaf.edu.cn/lsp/tcmsp.php), and previous EXD literatures [26–30, 35, 45, 46].

### Druggability analysis by LR, OB and DL properties

Lipinski’s rule (LR) [48] was used to identify druggable compounds according to the following criteria: molecular weight (MW) of not more than 500 Da (MW ≤500), chemical composition with no more than five hydrogen bond donors (H-bond donors ≤5), no more than 10 hydrogen bond acceptors (H-bond acceptors ≤10), and octanol–water partition coefficient, LogP, no >5 (LogP ≤ 5). A compound that does not satisfy at least two of the above conditions is less likely to be an orally active drug [49].

The phytochemical information of the compounds with their OB and DL properties were explored using the TCMSP database, which embed OBioavail 1.1 software for OB [50] and Tanimoto similarity software for DL [51]. The DL calculations in TCMSP database were based on the following formula [51]:

$$F(A,B) = A \times \frac{B}{{A^{2} + B^{2} - A \times B}}$$

where *A* is related to the molecular property of the target compound and *B* refers to the average molecular properties of all drugs from the Drugbank database (http://www.drugbank.ca/). A more detailed calculation of the DL index can be found in Tao et al. [51] and Wang et al. [52]. The thresholds used were OB ≥30% and DL index ≥0.18, as recommended by the TCMSP database. The thresholds were selected to efficiently identify bioactive compounds from the large pool of chemical compounds based on the following criteria: (1) the model obtained could be reasonably explained by previous pharmacological data and (2) the compound met the recommended mean DL index of 0.18 (the mean of DL index of 6,511 molecules from Drugbank database (2011) is 0.18) [51, 52].

### Identification of associated proteins and genes

The integrative efficacy of the identified constituents in EXD was determined by analyzing the chemical–protein and chemical–gene interactions obtained from the Search Tool for Interactions of Chemicals and Proteins (STITCH) database and Comparative Toxicogenomics Database (CTD), respectively. The STITCH 4.0 database (http://stitch.embl.de/) can be used to study potential interactions between 300,000 phytochemicals and 2.6 million proteins curated from 1,133 organisms [53]. In this database, the approximate probability of a predicted association for a chemical–protein interaction is determined by the confidence score, with a higher score indicating a stronger interaction (low confidence score ~0.2; medium confidence score ~0.5; high confidence score ~0.75; highest confidence score ~0.95, provided by STITCH 4.0 database). The CTD (http://ctd.mdibl.org/) is a publicly available research resource that includes more than 116,000 interactions between 9,300 chemicals and 13,300 genes [54]. Both databases were searched independently by two researchers to minimize any bias.

In order to identify the associated significant pathways, proteins with a chemical–protein interaction confidence score ≥0.5 were selected for the enrichment analysis by JEPETTO with the KEGG database, a Java-based Cytoscape 3.01 plugin [43]. For studying the gene-associated diseases, the genes were firstly ranked by frequency of occurrence of the chemical–gene interactions, and then the genes with gene frequency ≥1.67 were chosen for the enrichment analysis by Visualization and Integrated Discovery (DAVID) Bioinformatics Resources 6.7 (http://david.abcc.ncifcrf.gov/).

## Results

### Compounds in EXD

Eight hundred and ninety-five phytochemicals were collected from the six herbs in EXD. From the TCM Database@Taiwan, 203 compounds were identified, comprising 29 in HE, 44 in RC, 38 in RMO, 56 in RAS, seven in CPC, and 29 in RA. From the TCMSP database, 646 compounds were identified, comprising 130 in HE, 78 in RC, 174 in RMO, 125 in RAS, 58 in CPC, and 81 in RA. 46 phytochemicals from previous studies in the literature [26–30, 35, 45, 46], comprising 15 in HE, one in RC, five in ROM, five in RAS, 14 in CPC, 5 in RA, and one in EXD (specific herbs unknown). Finally, a total of 721 phytochemicals were identified in EXD after removing overlapping/duplicate compounds from the database s and the literature (Additional file 1).

### Identifying druggable compounds by LR, OB, and DL predictions

Of the 150 compounds from HE, 75 (50%) compounds were identified based on LR, 23 (15.3%) had OB ≥30% and DL index ≥0.18, and only 17 (11.3%) satisfied all criteria. Of the 104 compounds from RC, 29 (27.9%) passed LR, seven (6.7%) had OB ≥30% and DL index ≥0.18, and only four (3.8%) satisfied all criteria. Of the 189 compounds from RMO, 125 (66.1%) passed LR, 20 (10.6%) had OB ≥30% and DL index ≥0.18, and only 12 (6.3%) satisfied all criteria. Of the 173 compounds from RAS, 131 (75.7%) passed LR, five (2.9%) had OB ≥30% and DL index ≥0.18, and only three (1.7%) satisfied all criteria. Of the 63 compounds from CPC, 43 (68.3%) passed LR, 28 (44.4%) had OB ≥30% and DL index ≥0.18, and only 19 (30.2%) satisfied all criteria. Of the 81 compounds from RA, 45 (55.6%) passed LR, 15 (18.5%) had OB ≥30% and DL index ≥0.18, and only 11 (13.6%) satisfied all criteria (Table 1). The physicochemical properties of anemarsaponin BII from EXD reported in the literature (specific herbs unknown) did not pass LR. Overall, 66 compounds passed LR and had OB ≥30% and DL index ≥0.18. A total of 63 compounds were obtained after removing the duplicate compounds (Table 2).

**Table 1:** Compounds in EXD satisfying LR, OB ≥30% and DL ≥0.18

	Herbs					
	HE	RC	RMO	RAS	CPC	RA
Number of compounds	150	104	189	173	63	81
Compounds (percentage) passing LR	75 (50.0%)	29 (27.9%)	125 (66.1%)	131 (75.7%)	43 (68.3%)	45 (55.6%)
Compounds (percentage) with OB ≥30% and DL ≥0.18	23 (15.3%)	7 (6.7%)	20 (10.6%)	5 (2.9%)	28 (44.4%)	15 (18.5%)
Compounds (percentage) satisfying LR, OB ≥30% and DL ≥0.18	17 (11.3%)	4 (3.8%)	12 (6.3%)	3 (1.7%)	19 (30.2%)	11 (13.6%)

*LR* Lipinski’s rule, *OB* oral bioavailability, *DL* drug-likeness index, *HE* Herba *Epimedium Brevicornum*, *RC* *Rhizoma Curculiginis* *Orchioides*, *RMO* Radix *Morindae Officinalis*, *RAS* Radix *Angelicae Sinensis*, *CPC* Cortex *Phellodendri Chinensis*, *RA* Rhizoma *Anemarrhenae asphodeloides*.

**Table 2:** The 63 bioactive compounds from HE, RC, RMO, RAS, CPC, and RA herbs and their corresponding molecular properties, OB and DL (20 of 63 bioactive compounds related to 34 significant pathway- or 12 gene- associated with menopause)

Phytochemical	MW	AlogP	Hdon	Hacc	OB (%)	DL	Herb
1. DFV((2S)-7-hydroxy-2-(4-hydroxyphenyl)chroman-4-one)^b^	256.27	2.57	2	4	32.76	0.18	HE
2. Delta7-dehydrosophoramine	242.35	1.09	0	3	54.45	0.25	CPC
3. Alizarin-2-methylether	254.25	2.53	1	4	32.81	0.21	RMO
4. 1-Hydroxy-3-methoxy-9,10-anthraquinone	254.25	2.53	1	4	104.33	0.21	RMO
5. 1-Hydroxy-6-hydroxymethylanthracenequinone	254.25	1.94	2	4	81.77	0.21	RMO
6. Skimmianin (4,7,8-trimethoxyfuro[2,3-b]quinoline)	259.28	2.33	0	5	40.14	0.20	CPC
7. Magnograndiolide ((3aS,6R,6aR,9R,9aS,9bS)-6,9-dihydroxy-6,9-dimethyl-3-methylidene-3a,4,5,6a,7,8,9a,9b-octahydroazuleno[5,4-d]furan-2-one)	266.37	1.18	2	4	63.71	0.19	HE/CPC
8. Coumaroyltyramine (cis-*N*-p-Coumaroyltyramine)	283.35	2.88	3	4	112.9	0.20	RA
9. Kaempferol(3,5,7-trihydroxy-2-(4-hydroxyphenyl)chromen-4-one)^b^	286.25	1.77	4	6	41.88	0.24	HE/RA
10. Luteolin(2-(3,4-dihydroxyphenyl)-5,7-dihydroxychromen-4-one)^b^	286.25	2.07	4	6	36.16	0.25	HE
11. Rutaecarpine(Indolo(2′,3′:3,4)pyrido(2,1-b)quinazolin-5(7H)-one, 8,13-dihydro-(9CI))^b^	287.34	3.36	1	3	40.30	0.60	CPC
12. 8-(3-methylbut-2-enyl)-2-phenyl-chromone	290.38	4.99	0	2	48.54	0.25	HE
13. Dehydrotanshinone II A (1,6,6-trimethyl-7H-naphtho[5,6-g] [1] benzoxole-10,11-dione)	292.35	4.22	0	3	43.76	0.40	CPC
14. Chryseriol(5,7-dihydroxy-2-(4-hydroxy-3-methoxyphenyl)chromen-4-one)^b^	300.28	2.32	3	6	35.85	0.27	HE
15. Phellopterin(4-methoxy-9-(3-methylbut-2-enoxy)furo[3,2-g]chromen-7-one)^b^	300.33	3.64	0	5	40.19	0.28	CPC
16. Cnidilin(9-methoxy-4-(3-methylbut-2-enoxy)furo[3,2-g]chromen-7-one)^b^	300.33	3.64	0	5	32.69	0.28	RAS
17. Quercetin(2-(3,4-dihydroxyphenyl)-3,5,7-trihydroxychromen-4-one)^b^	302.25	1.50	5	7	46.43	0.28	HE
18. (*Z*)-3-(4-hydroxy-3-methoxy-phenyl)-*N*-[2-(4-hydroxyphenyl)ethyl]acrylamide	313.38	2.86	3	5	118.35	0.26	RA
19. 1,6-dihydroxy-5-methoxy-2-(methoxymethyl)-9,10-anthraquinone	314.31	2.06	2	6	104.54	0.34	RMO
20. Hippeastrine(Lycorenan-7-one, 5-hydroxy-1-methyl-9,10-(methylenebis(oxy))-, (5alpha)-)	315.35	1.17	1	6	51.65	0.62	RA
21. Coptisine(6,7-dihydro-bis(1,3)benzodioxolo (5,6-a:4′,5′-g)quinolizinium)	320.34	3.25	0	4	30.67	0.86	CPC
22. 1,2-bis(4-hydroxy-3-methoxyphenyl)propan-1,3-diol	320.37	1.69	4	6	52.31	0.22	HE
23. 2-Hydroxy-1,5-dimethoxy-6-(methoxymethyl)-9,10-anthraquinone	328.34	2.31	1	6	95.85	0.37	RMO
24. 2-Hydroxy-1,8-dimethoxy-7-methoxymethylanthracenequinone	328.34	2.31	1	6	112.30	0.37	RMO
25. Americanin A^a^	328.34	2.30	3	6	46.71	0.35	RMO
26. C-Homoerythrinan, 1,6-didehydro-3,15,16-trimethoxy-, (3.beta.)	329.48	2.89	0	4	39.14	0.49	HE
27. 1,5,7-Trihydroxy-6-methoxy-2-methoxymethylanthracenequinone	330.31	1.79	3	7	80.42	0.38	RMO
28. (2R,3S)-(+)-3′,5-Dihydroxy-4,7-dimethoxydihydroflavonol	332.33	1.99	3	7	77.24	0.33	RMO
29. 2-Hydroxyethyl 5-hydroxy-2-(2-hydroxybenzoyl)-4-(hydroxymethyl)benzoate	332.33	1.41	4	7	62.32	0.26	RMO
30. Chelerythrine^a,b^	332.37	4.29	0	4	34.18	0.78	CPC
31. Worenine^a^	334.37	3.73	0	4	45.83	0.87	CPC
32. Yinyanghuo C(2-(2,2-dimethylchromen-6-yl)-5,7-dihydroxychromen-4-one)	336.36	3.39	2	5	45.67	0.50	HE
33. Berberine^a,b^	336.39	3.45	0	4	36.86	0.78	CPC
34. Isocorypalmine ((13aS)-5,8,13,13a-tetrahydro-3,9,10-trimethoxy-6H-Dibenzo[a,g]quinolizin-2-ol)	341.44	3.35	1	5	35.77	0.59	CPC
35. Yinyanghuo E (5,7-dihydroxy-2-(8-hydroxy-2,2-dimethylchromen-6-yl)chromen-4-one)	352.36	3.12	3	6	51.63	0.55	HE
36. Palmatine(Palmatine chloride is another name in TCMSP database)^b^	352.44	3.65	0	4	64.6	0.65	CPC
37. Fumarine(7-methyl-6,8,9,16-tetrahydrobis[1, 3]benzodioxolo[4,5-c:5′,6′-g]azecin-15(7H)-one)^b^	353.40	2.95	0	6	59.26	0.83	CPC
38. Cavidine(9-dimethoxy-6-methyl-6,6a,11,14-tetrahydro-8,12H-benzo(a)-1,3-benzodioxolo(4,5-g)quinolizine)	353.45	3.72	0	5	35.64	0.81	CPC
39. 8-Isopentenyl-kaempferol^b^	354.38	3.63	4	6	38.04	0.39	HE
40. Anhydroicaritin(3,5,7-trihydroxy-2-(4-methoxyphenyl)-8-(3-methylbut-2-enyl)chromen-4-one)^b^	368.41	3.88	3	6	45.41	0.44	HE/RA
41. Suchilactone^a^	368.41	3.73	0	6	57.52	0.56	RAS
42. 6-Hydroxy-11,12-dimethoxy-2,2-dimethyl-1,8-dioxo-2,3,4,8-tetrahydro-43.1H-isochromeno[3,4-h]isoquinolin-2-ium	370.41	2.75	1	6	60.64	0.66	HE
43. Ohioensin-A^a^	372.39	3.57	3	5	38.13	0.76	RMO
44. Phellavin_qt^a^	374.42	2.51	5	7	35.86	0.44	CPC
45. Olivil^a^	376.44	1.68	4	7	62.23	0.41	HE
46. Jatrorrhizine^a,b^	380.5	4.44	1	4	30.44	0.75	CPC
47. Stigmasterol((3S,8S,9S,10R,13R,14S,17R)-17-[(2R,5S)-5-ethyl-6-methylhept-3-en-2-yl]-10,13-dimethyl-2,3,4,7,8,9,11,12,14,15,16,17-dodecahydro-1H-cyclopenta[a]phenanthren-3-ol)^b^	412.77	7.64	1	1	43.83	0.76	RC
48. Diosgenin((3β,25*R*)-spirost-5-en-3-ol)^b^	414.69	4.63	1	3	80.88	0.81	RA
49. ZINC03982454((3R,8S,9S,10R,13R,14R,17R)-17-[(2R,5S)-5-ethyl-6-methylheptan-2-yl]-10,13-dimethyl-2,3,4,7,8,9,11,12,14,15,16,17-dodecahydro-1H-cyclopenta[a]phenanthren-3-ol)	414.79	8.08	1	1	36.91	0.76	RC
50. Beta-sitosterol(17-(5-Ethyl-6-methylheptan-2-yl)-10,13-dimethyl-2,3,4,7,8,9,11,12,14,15,16,17-dodecahydro-1*H*-cyclopenta[*a*]phenanthren-3-ol)^b^	414.79	8.08	1	1	36.91	0.75	RC
51. Timosaponin B III_qt^a^	416.71	4.77	2	3	35.26	0.87	RA
52. Anemarsaponin C_qt^a^	416.71	4.97	2	3	35.50	0.87	RA
53. Phyllanthin(4-[(2S,3S)-3-[(3,4-dimethoxyphenyl)methyl]-4-methoxy-2-(methoxymethyl)butyl]-1,2-dimethoxybenzene)	418.58	4.11	0	6	33.31	0.42	RAS
54. Yinyanghuo A^a^	420.49	4.20	3	6	56.96	0.77	HE
55. Cycloartenol (9beta,19-Cyclo-24-lanosten-3beta-ol)^b^	426.80	7.55	1	1	38.69	0.78	RC
56. Anemarsaponin F_qt^a^	432.71	3.92	2	4	60.06	0.79	RA
57. Asperglaucide(aurantiamide acetate)	444.57	4.02	2	6	58.02	0.52	RA
58. Anemarsaponin E_qt^a^	448.76	4.53	2	4	30.67	0.86	RA
59. Obacunone^a,b^	454.56	2.68	0	7	43.29	0.77	CPC
60. Icariside A7((2R,3S,4S,5R,6S)-2-(hydroxymethyl)-6-(7-hydroxy-3,4,6-trimethoxyphenanthren-2-yl)oxyoxane-3,4,5-triol0	462.49	1.16	5	10	31.91	0.86	HE
61. Hispidone^a^	472.78	4.46	2	4	36.18	0.83	CPC
62. Kihadanin A^a^	486.56	1.76	1	9	31.60	0.70	CPC
63. Isoprincepin^a^	494.53	2.52	5	9	49.12	0.77	RMO

*HE* herba *Epimedium Brevicornum*, *RC* Rhizoma *Curculiginis Orchioides*, *RMO* Radix *Morindae Officinalis*, *RAS* Radix *Angelicae Sinensis*, *CPC* Cortex *Phellodendri Chinensis*, *RA* Rhizoma *Anemarrhenae Asphodeloides*.

^a^IUPAC name were not provided in database.

^b^20 EXD bioactive compounds related to 34 significant pathway- or 12 gene- associated with menopause.

### Revealing the significant pathways and gene-associated diseases

Overall, 155 of the 721 compounds from EXD were found to have 2,656 chemical–protein interactions. After removing the overlapping/duplicate information, 1,963 associated proteins were obtained (Additional file 2). 1,824 of 1,963 proteins with a confidence score exceeding 0.5 were obtained. After enrichment analysis of 1,824 associated proteins, XD-scores and q values of pathways have been obtained. The XD-score is relative to the average distance to all pathways and represents a deviation from the average distance [43]. A larger positive XD-score indicates a stronger association between the inputted associated proteins and molecular interaction network of pathways. The q value determines the significance of the overlap (Fisher’s exact test) between the input information and the pathways. The enrichment algorithm analysis (graph-based statistic) of XD-score and q-value revealed that the threshold value of XD-score in this study was 0.67, therefore there are 34 pathways significantly associated with input set of proteins (Table 3).

**Table 3:** The 34 significant pathways found by JEPETTO (Cytoscape plugin) with KEGG database

Pathway	XD-score	q value	Overlap/size
Linoleic acid metabolism	3.148	0.000	10/11
Citrate cycle (TCA cycle)	3.057	0.000	21/26
Propanoate metabolism	1.899	0.000	11/19
Arachidonic acid metabolism	1.865	0.000	17/26
PPAR signaling pathway	1.794	0.000	22/39
Tyrosine metabolism	1.475	0.001	9/17
Retinol metabolism	1.474	0.009	6/12
Bladder cancer	1.463	0.000	17/38
Ether lipid metabolism	1.370	0.002	8/16
Metabolism of xenobiotics by cytochrome P450	1.313	0.001	10/20
Adipocytokine signaling pathway	1.248	0.000	24/57
Drug metabolism: cytochrome P450	1.240	0.003	8/17
Fatty acid metabolism	1.096	0.000	12/26
Pyruvate metabolism	1.096	0.000	12/26
One carbon pool by folate	1.057	0.018	5/10
Glyoxylate and dicarboxylate metabolism	1.057	0.018	5/10
Fc epsilon RI signaling pathway	1.040	0.000	23/65
Pancreatic cancer	1.013	0.000	25/70
Steroid hormone biosynthesis	0.990	0.027	6/15
GnRH signaling pathway	0.969	0.000	32/83
Beta-Alanine metabolism	0.924	0.007	7/15
Prostate cancer	0.913	0.000	32/84
Tryptophan metabolism	0.903	0.000	12/26
Long-term depression	0.893	0.000	23/57
Toll-like receptor signaling pathway	0.883	0.000	32/90
NOD-like receptor signaling pathway	0.879	0.000	21/59
Biosynthesis of unsaturated fatty acids	0.875	0.027	5/11
Riboflavin metabolism	0.875	0.027	5/11
VEGF signaling pathway	0.872	0.000	24/62
Glycerophospholipid metabolism	0.829	0.002	13/35
Type II diabetes mellitus	0.814	0.010	13/43
Chagas disease	0.804	0.000	36/99
Selenoamino acid metabolism	0.763	0.049	6/17
Renal cell carcinoma	0.708	0.000	24/68

**Figure 2:**
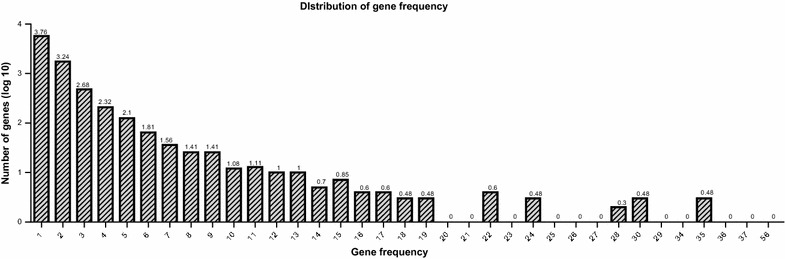
Gene frequency of the associated genes of 210 compounds.

In total, 210 of the 721 compounds from EXD were found to have 14,893 compound–gene interactions with 8,536 associated genes in the CTD (Additional file 3). Subsequently, the 8,536 genes were ranked according to their frequency of occurrence. The number of genes fell abruptly when the frequency of occurrence was small (gene frequency ≤8; Figure 2). Subsequently, the number of genes became stabilized for gene frequencies between 10 and 19. However, the number of genes with gene frequencies ≥20 was quite small. Genes with gene frequencies below the average of 1.74 were removed to reduce the number of redundant genes. After that, the remaining 2,183 genes were used to conduct the gene enrichment analysis by the DAVID platform. The “GENETIC_ASSOCIATION_DB_DISEASE_CLASS” was selected as the annotation category to search for the significant diseases associated with the input genes, which was statistically verified by Fisher’s exact test using the DAVID platform [47]. *P* ≤ 0.01 indicated significant association or enrichment with the related items. After removing nonspecific diseases, 12 classes of diseases were found to be highly associated with the input genes (Tables 4 and 5). Most of these diseases were related to menopause, such as aging, reproduction, cancer, cardiovascular diseases, and neurological diseases [55–58].

**Table 4:** Chemical–protein interactions and related significant signaling pathways

Herb	Compound	Protein	Pathway
HE	Emodin (1,3,8-trihydroxy-6-methylanthracene-9,10-dione)	HSD11B1	Steroid hormone biosynthesis
RMO	Alcool methylique	SULT2B1	Steroid hormone biosynthesis
CPC	Phenanthrene(TCMSP don’t record IUPAC name)	CYP1A1	Steroid hormone biosynthesis
RMO	Caffeic acid ((*Z*)-3-(3,4-dihydroxyphenyl)prop-2-enoic acid)	COMT	Steroid hormone biosynthesis
HE	Quercetin(2-(3,4-dihydroxyphenyl)-3,5,7-trihydroxychromen-4-one)	CYP19A1	Steroid hormone biosynthesis
HE	Quercetin-3-*O*-glucoside	CYP1B1	Steroid hormone biosynthesis
HE	Apigenin(5,7-dihydroxy-2-(4-hydroxyphenyl)chromen-4-one)	PTK2	VEGF signaling pathway
RMO	Hemo-sol((4R)-1-methyl-4-prop-1-en-2-ylcyclohexene)	HRAS	VEGF signaling pathway
HE/RA	Kaempferol(3,5,7-trihydroxy-2-(4-hydroxyphenyl)chromen-4-one)	MAPK1/AKT1/SRC	VEGF signaling pathway
RMO	Esculetin(6,7-dihydroxychromen-2-one)	MAPK3	VEGF signaling pathway
RMO	Citric acid(2-hydroxypropane-1,2,3-tricarboxylic acid)	KRAS	VEGF signaling pathway
HE	Oleanolic acid((3-beta)-3-Hydroxyolean-12-en-28-oic acid)	MAPK14	VEGF signaling pathway
RAS	Adenine(7H-purin-6-amine)	PTGS2	VEGF signaling pathway
HE	Emodin(1,8-dihydroxy-3-(hydroxymethyl)anthracene-9,10-dione)	VEGFA	VEGF signaling pathway
HE	Luteolin(2-(3,4-dihydroxyphenyl)-5,7-dihydroxychromen-4-one)	PIK3CB	VEGF signaling pathway
RA	3,5,7-Trihydroxy-4′-methoxyl-8-prenylflavone-3-*O*-rhamnopyranoside	CASP9	VEGF signaling pathway
RC/RMO	Myristic acid	PPP3CB/PPP3CA	VEGF signaling pathway
RC/RMO/RAS	Palmitic acid	NOS3	VEGF signaling pathway
RAS	Lecithin	PLA2G4A	VEGF signaling pathway
HE/RMO	Lauric acid	PIK3CA	VEGF signaling pathway
RA	Chinoinin(1,3,6,7-tetrahydroxy-2-[(2S,3R,4R,5S,6R)-3,4,5-trihydroxy-6-(hydroxymethyl)oxan-2-yl]xanthen-9-one)	PLA2G2A	VEGF signaling pathway
RAS	4-Methoxybenzoic acid	PLA2G1B	VEGF signaling pathway
RMO	Nonanoic acid	NFAT5/NFATC3	VEGF signaling pathway
RMO	(9Z,12Z)-octadeca-9,12-dienoic acid	PLA2G10/PLA2G5	VEGF signaling pathway
RC/RMO	Myristic acid	PPP3CC	VEGF signaling pathway

**Table 5:** The 12 disease classes highly associated with input genes

Disease class	Number of input genes involved in the disease	Input genes/total genes involved in the disease (%)	*P* value
Cancer	384	14.5	9.4E−28
Cardiovascular	342	12.9	2.8E−25
Aging	79	3.0	8.7E−17
Reproduction	133	5.0	1.3E−11
Renal	100	3.8	2.5E−10
Neurological	247	9.3	1.9E−7
Infection	142	5.4	2.0E−7
Psychological	238	9.0	2.9E−7
Immune	316	11.9	1.4E−5
Hematological	70	2.6	1.4E−4
Vision	85	3.2	4.4E−4
Developmental	105	4.0	6.4E−3

### Identifying twenty bioactive compounds related to menopause with following the druggability prediction

Eighteen of the 155 compounds that have 2,656 chemical–protein interaction, followed the Lipinski’s Rule with OB ≥30% and DL index ≥0.18. Thirteen of the 210 compounds that have compound–gene interactions interaction, followed the Lipinski’s Rule with OB ≥30% and DL index ≥0.18. Finally, 11 compounds has been identified related to both chemical–gene and chemical–protein interaction and followed the druglikeness prediction. Moreover, 20 compounds related to 34 significant pathway- or 12 gene- associated with menopause have been identified (Table 3).

## Discussion

The actions of bioactive compounds in EXD were investigated by combining a drug prediction method with an enrichment analysis using information from bioinformatics databases at the gene and protein levels. For example, candidate compounds such as berberine, palmatine, and jatrorrhizine, which we identified using our drug prediction method, have been shown to exhibit extensive pharmacological activities [59, 60]. From the enrichment analysis based on the available information for compound–protein and compound–gene interactions of EXD, we identified the most significantly related pathways and gene-associated disease, including pathways related to endocrine [35], VEGF [61], lipid metabolism [62] and anti-inflammatory [34]. Their pharmacological association with EXD were in line with previous publications [34, 35, 61, 62].

**Figure 3:**
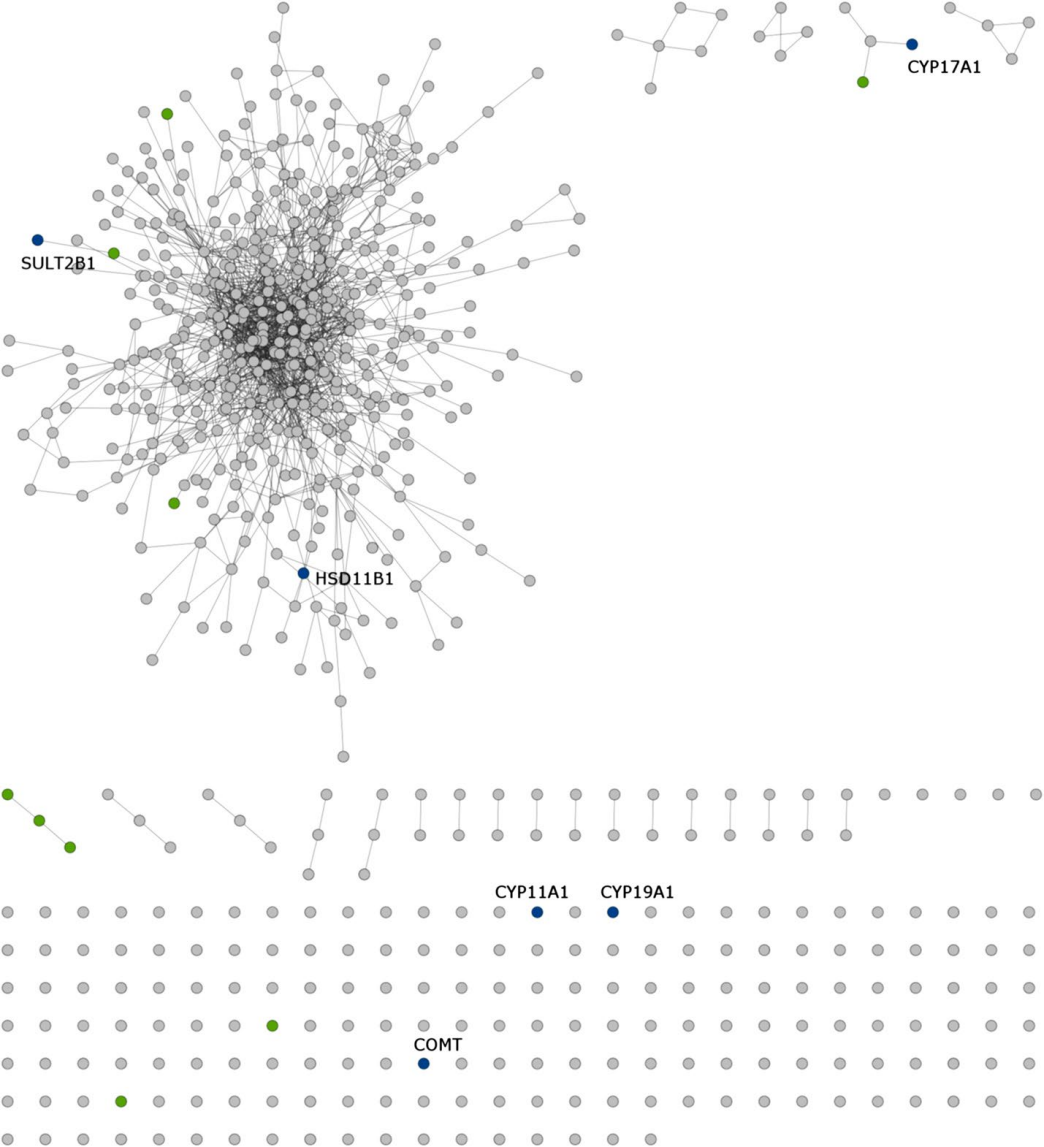
Chemical–protein interactions related to steroid hormone biosynthesis pathways. The *grey* color represents genes in the target set,* green* relates to the steroid hormone biosynthesis pathway, *blue* (labeled) is the overlap between the related pathway and the input protein set.

Several pathways involved the endocrine have also been identified, such as steroid hormone biosynthesis, GnRH signaling pathway, and adipocytokine signaling pathway, covering the previous finding of our group to promote estradiol biosynthesis in animal study [35]. For the steroid hormone biosynthesis signaling pathways, the EXD compound, quercetin, promoted the expression of aromatase (CYP19A1), which is the enzyme for estrogen biosynthesis [63]. This compound also met the druggability criteria. Other important overlapping proteins were HSD11B1, SULT2B1, CYP1A1, COMT, and CYP1B1 (Figure 3).

**Figure 4:**
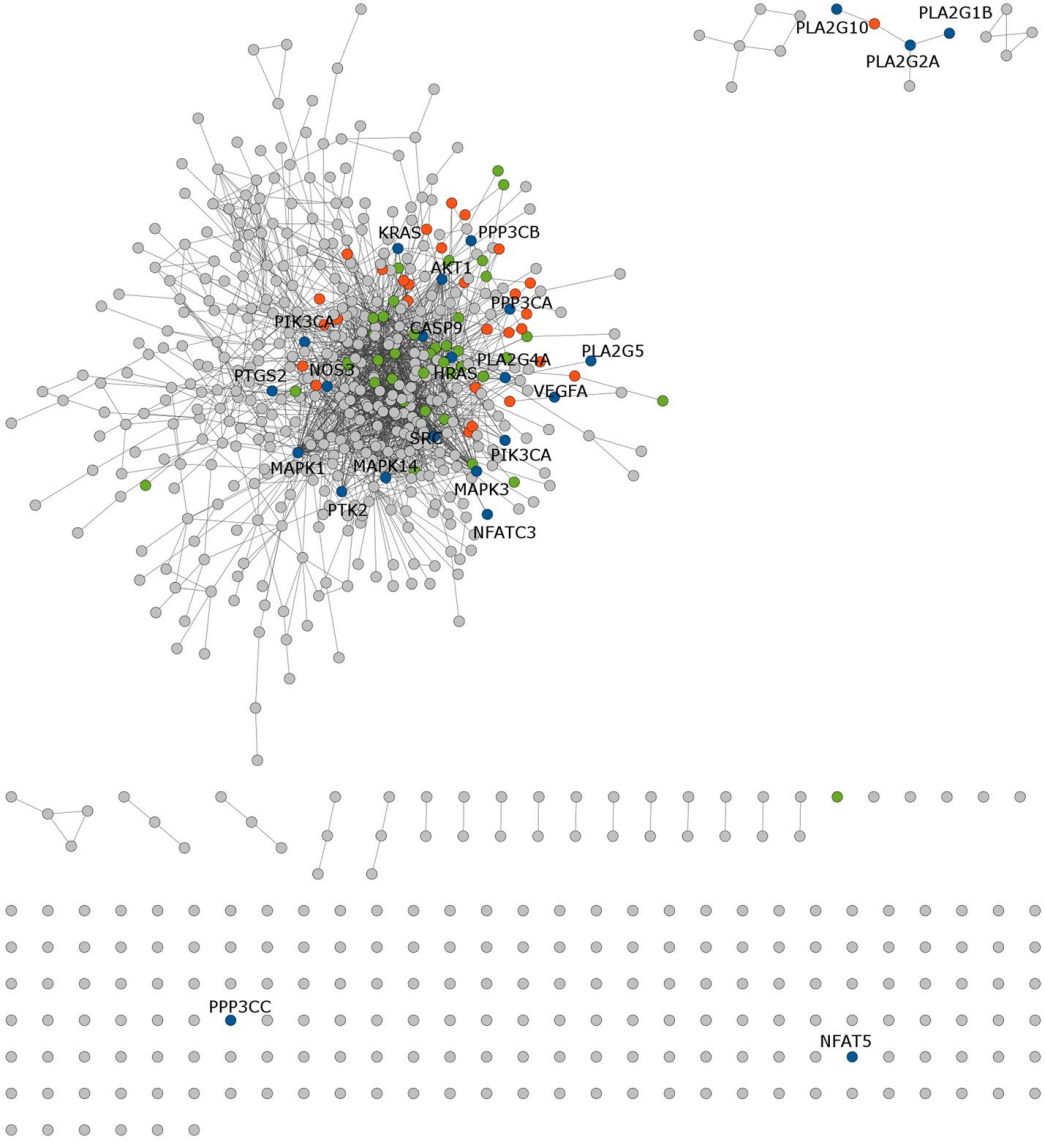
Chemical–protein interactions related to the VEGF signaling pathway. The *grey* color represents genes in the target set,* green* relates to the VEGF pathway, *blue* (labeled) is the overlap between the related pathway and the input protein set. The *orange* is the expansion of their pathways.

For the VEGF signaling pathways, VEGFA protein was involved in the antiangiogenic ability of EXD from our previous study [61]. The anti-cancer effect of EXD compound interact with VEGFA, emodin, has been reported [64]. Other interacting proteins of significance were PTK2, HRAS, MAPK1, AKT1, SRC, MAPK3, KRAS, MAPK14, PTGS2, PIK3CB, CASP9, PPP3CB, PPP3CA, NOS3, PLA2G4A, PIK3CA, PLA2G2A, PLA2G1B, NFAT5, NFATC3, PLA2G10, PLA2G5, and PPP3CC (Figure 4). The steroid hormone biosynthesis and VEGF signaling pathways were selected for further analysis in the present study (Table 5).

For the lipid metabolism, EXD associated-pathways related to linoleic acid metabolism, fatty acid metabolism, unsaturated fatty acid biosynthesis, glycerophospholipid metabolism, arachidonic acid metabolism, and PPAR were identified [65–69]. Besides, our previous study found that EXD could improve the lipid profile in cardiovascular disease [62].

While a previous study showed EXD to have anti-inflammatory activity [34], the present study suggested the pathways to include the Toll-like receptor signaling pathway, NOD-like receptor signaling pathway, and Fc epsilon RI signaling pathway [70–72]. This findings were consistent with previous studies on EXD antimetastatic activity in a human ovarian cancer model [73] and its antiangiogenic properties [61].

Compound–compound interactions were not considered in this study because the available databases could only provide limited information for the six individual herbs. The information of the databases did not cover the new compounds synthesized by chemical reactions during the decoction of EXD’s ingredients, which will be confirmed by liquid chromatograph couple with mass spectrometry in further study. The ranking of the compound–gene and compound–protein interaction information was based on published evidence, but qualify of this evidence still needs extensive assessment. This study exemplified how to screen and identify bioactive compounds in CHFs.

## Conclusions

Twenty compounds were identified by network pharmacology as potential effective ingredients of EXD for menopause with acceptable oral bioavailability and druggability.

## Additional files

**Additional file 1.** Information on 721 identified compounds in EXD.
**Additional file 2.** Information on chemical-protein interaction pertaining to 155 compounds from EXD.
**Additional file 3.** Information on chemical-gene interaction pertaining to 210 compounds from EXD.

## Abbreviations

EXD: *Erxian decoction*
CHFs: Chinese herbal formulas
TCMSP: traditional Chinese medicine systems pharmacology
STITCH: search tool for interactions of chemicals and proteins
CTD: The Comparative Toxicogenomics Database
LH: luteinizing hormone
FSH: follicle-stimulating hormone
HRT: hormone replacement therapy
CAM: complementary and alternative medicine
CM: Chinese medicine
OB: oral bioavailability
DL: drug-likeness
DAVID: The Database for Annotation, Visualization and Integrated Discovery
E2: estradiol
HSD11B1: hydroxysteroid (11-beta) dehydrogenase 1
SULT2B1: sulfotransferase family cytosolic 2B member 1
COMT: catechol-*O*-methyltransferase
CYP1A1: cytochrome P450, family 1, subfamily A, polypeptide 1
CYP1B1: cytochrome P450, family 1, subfamily B, polypeptide 1
CYP19A1: cytochrome P450, family 19, subfamily A, polypeptide 1
VEGF: vascular endothelial growth factor
GnRH: gonadotropin-releasing hormone
PTK2: protein tyrosine kinase 2
HRAS: Transforming protein p21
AKT1: RAC-alpha serine/threonine-protein kinase
MAPK1: mitogen-activated protein kinase 1
MAPK3: mitogen-activated protein kinase 3
SRC: SRC proto-oncogene, non-receptor tyrosine kinase
KRAS: GTPase KRas
CASP9: caspase-9
NOS3: nitric oxide synthase 3
PIK3CA: phosphatidylinositol-4,5-bisphosphate 3-kinase, catalytic subunit alpha isoform
PIK3CB: phosphatidylinositol-4,5-bisphosphate 3-kinase catalytic subunit beta isoform
PLA2G1B: phospholipase A2
PLA2G2A: membrane-associated phospholipase A2
PLA2G4A: cytosolic phospholipase A2
PLA2G5: calcium-dependent phospholipase A2
PLA2G10: group 10 secretory phospholipase A2
NFAT5: nuclear factor of activated T-cells 5
NFATC3: nuclear factor of activated T-cells, cytoplasmic 3
PPP3CA: protein phosphatase 3, catalytic subunit alpha isoform
PPP3CB: serine/threonine-protein phosphatase 2C catalytic subunit beta isoform
PPP3CC: serine/threonine-protein phosphatase 2B catalytic subunit gamma isoform
